# Targeting senescence as an anticancer therapy

**DOI:** 10.1002/1878-0261.13312

**Published:** 2022-09-23

**Authors:** Laura Bousset, Jesús Gil

**Affiliations:** ^1^ MRC London Institute of Medical Sciences (LMS) UK; ^2^ Faculty of Medicine, Institute of Clinical Sciences (ICS) Imperial College London UK

**Keywords:** cellular senescence, chemotherapy, oncogene‐induced senescence, radiotherapy, senolytics, therapy‐induced senescence

## Abstract

Cellular senescence is a stress response elicited by different molecular insults. Senescence results in cell cycle exit and is characterised by multiple phenotypic changes such as the production of a bioactive secretome. Senescent cells accumulate during ageing and are present in cancerous and fibrotic lesions. Drugs that selectively kill senescent cells (senolytics) have shown great promise for the treatment of age‐related diseases. Senescence plays paradoxical roles in cancer. Induction of senescence limits cancer progression and contributes to therapy success, but lingering senescent cells fuel progression, recurrence, and metastasis. In this review, we describe the intricate relation between senescence and cancer. Moreover, we enumerate how current anticancer therapies induce senescence in tumour cells and how senolytic agents could be deployed to complement anticancer therapies. “One‐two punch” therapies aim to first induce senescence in the tumour followed by senolytic treatment to target newly exposed vulnerabilities in senescent tumour cells. “One‐two punch” represents an emerging and promising new strategy in cancer treatment. Future challenges of “one‐two punch” approaches include how to best monitor senescence in cancer patients to effectively survey their efficacy.

## Graphical abstract

Conventional cancer therapies induce senescence. While senescence contributes to therapy outcome, accumulation of senescent cells can promote cancer progression, metastasis and therapy resistance. One‐two punch approaches aim to combine cancer therapies with senolytics to remove senescent tumour cells. Here, we review senescence in the context of cancer and discuss future challenges of senotherapies for cancer treatment.

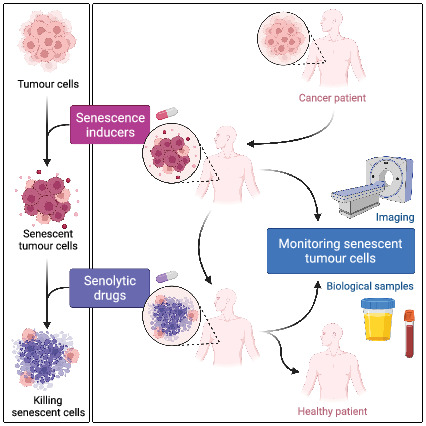

**Abbreviations**
17‐AAG: 17‐allylamino‐17‐demethoxygeldanamycin
17‐DMAG: 17‐dimethylaminoethylamino‐17‐demethoxygeldanamycin
ADCC: antibody‐dependent cell‐mediated cytotoxicity
AML: acute myeloid leukaemia
AMPK: AMP‐activated protein kinase
AURK: aurora kinase
BET: bromodomain and extraterminal domain
BETd: bromodomain and extraterminal domain degrader
CAR T cell: chimeric antigen receptor T cell
CDK: cyclin‐dependent kinase
CG: cardiac glycoside
cGAMP: cGMP‐AMP
cGAS: cyclic GMP‐AMP synthase
CML: chronic myeloid leukaemia
D +Q: dasatinib and quercetin
DDR: DNA damage response
DNA‐SCARS: DNA segments with chromatin alterations reinforcing senescence
DNMT: DNA methyltransferase
DRI: D‐Retro inverso
EMT: epithelial‐mesenchymal transition
GMD: galactose‐modified duocarmycin
HCC: hepatocellular carcinoma
HDAC: histone deacetylase
HNSCC: head and neck squamous cell carcinoma
HR: hormone receptor
HSP: heat shock protein
HUVEC: human umbilical vein endothelial cell
ICI: immune checkpoints inhibitors
ICSA: International Cell Senescence Association
IKK: IkB kinase
KAT: histone lysine acetyltransferase
LSEC: liver sinusoidal endothelial cell
MEF: mouse embryonic fibroblast
MMP: matrix metalloproteinase
NK: natural killer
NSCLC: non‐small cell lung cancer
OIS: oncogene‐induced senescence
OSCC: oral squamous cell carcinoma
PARP: poly(ADP‐ribose) polymerase
PARPi: PARP inhibitor
PASP: p21‐activated secretory phenotype
PDAC: pancreatic ductal adenocarcinoma
PI3K: phosphatidylinositol tri‐phosphate
PICS: PTEN loss‐induced cellular senescence
PIN: prostate intraepithelial neoplasia
PLK: polo‐like kinase
PROTAC: proteolysis targeting chimera
ROS: reactive oxygen species
RTK: receptor tyrosine kinase
SAHA: suberoyanilide hydroxamic acid
SASP: senescence‐associated secretory phenotype
SA‐β‐gal: senescence‐associated β‐galactosidase
STING: stimulator of interferon genes
suPAR: soluble form of uPAR
TIS: therapy‐induced senescence
TKI: tyrosine kinase inhibitor
TME: tumour microenvironment
uPAR: urokinase plasminogen activator receptor

## Introduction

During their lifetime, cells are subjected to a variety of damages. Depending on the nature and strength of those damages, cells can repair them. When this is not possible, they activate death signalling pathways to avoid the impact that damage cells could have on tissue homeostasis. An alternative to trigger cell death is cellular senescence. Cellular senescence is a highly stable cell cycle arrest initiated in response to a variety of stress signals to prevent the replication of old, damaged or preneoplastic cells [1]. The implementation of the senescence growth arrest depends on the activation of the p21^CIP1/WAF1^/p53 and p16 ^INK4A^/RB tumour suppressor pathways [2].

Besides undergoing a highly‐stable cell cycle arrest, senescent cells reprogram their metabolism, suffer structural changes, epigenetic modifications and macromolecular (DNA, proteins, lipids) damage and induce a bioactive secretome, termed the senescence‐associated secretory phenotype (SASP) [1, 3, 4, 5]. How the SASP is regulated has been reviewed elsewhere [6, 7]. Briefly, the SASP is linked to the DNA damage response (DDR) [8] and its induction requires sensing macromolecular damage by elements of the innate immune pathways (such as cGAS/STING or the inflammasome) [9, 10, 11]. Eventually, these signals are integrated and activate key factors such as mTOR [12, 13], GATA4 [14] or p38 MAPK [15]. Ultimately, the sensors and mediators of the SASP induction converge to the activation of key transcriptional factors such as NF‐κB and C/EBPβ that control the expression of key inflammatory factors such as IL‐1β, IL‐6 and IL‐8 [16, 17, 18]. The secretion of these factors creates a feedback loop that reinforce the SASP phenotype [19].

Senescence was initially described in 1961 by Hayflick et al. [20] who revealed that normal cells stop to replicate after a finite number of passages. Nowadays, we refer to this phenomenon as replicative senescence. It is attributed to the end replication problem that shortens telomere, provokes persistent DNA damage and induces the downstream activation of senescence [21, 22]. Importantly, other insults such as oncogene activation, genotoxic and oxidative stress or mitochondrial defects also lead to senescence independently of telomeric shortening (referred collectively as premature senescence) [23]. For example, expression of oncogenic RAS in primary cells is associated with a permanent cell cycle arrest phenotypically indistinguishable from cellular senescence (oncogene‐induced senescence, OIS) [24].

Importantly, senescence plays multiple roles in health and disease. Transient senescence activation is part of normal tissue development through embryogenesis (developmental senescence) [25, 26] to adulthood (where it maintains tissue homeostasis) [27]. Induction of senescence limits the replication of damaged cells and elicits their elimination by the immune system in a SASP‐dependent fashion. In this context, acute senescence is beneficial, and for example contributes to limit fibrosis [28] and cancer initiation [29]. However, when senescent cells linger in a tissue they often play detrimental roles, contributing to ageing and many diseases, including paradoxically cancer progression [30].

Identifying senescent cells is key to better understand their roles *in vivo*. This is often achieved by assaying for senescence‐associated β‐galactosidase activity (SA‐β‐gal), upregulated in senescent cells due to their increase in lysosomal biogenesis [31]. Other markers such as expression of the cell cycle regulators p16^INK4A^, reduced levels of Lamin B1, absence of proliferation or induction of SASP components are also used to identify senescence. Due to the heterogeneity of senescence, a combination of those markers is needed to assess senescence, as summarised in the consensus position from the International Cell Senescence Association (ICSA) [1].

## Senescent cells in tumours

Different types of senescent cells are present in the tumour microenvironment (TME) during cancer initiation, progression and in response to therapy. Many preneoplastic lesions, including lung adenomas [32], melanocytic nevi [33], lymphomas [34] or prostate intraepithelial neoplasia (PIN) [35] are enriched in senescent cells. This is because activation of oncogenes (e.g., RAS in lung or BRAF in nevi) or loss of tumour suppressor (e.g., PTEN in the prostate) induces senescence, what restrains tumour progression. Another contributor to senescence induction in the context of tumorigenesis are anticancer treatments, as radiotherapy, conventional chemotherapy and some targeted therapies: that cause so‐called therapy‐induced senescence (TIS) in the tumour cells [36]. Cancer therapies can also induce senescence in cells other than the tumour cells. Indeed, induction of senescence in normal tissues has been suggested to cause some of the side effects associated with chemotherapy [37]. Finally, other cells in the TME might also undergo senescence [38]. Stromal senescent cells are an emerging factor contributing to tumorigenesis and promoting cancer drug resistance [39, 40]. Senescent cells in the TME can also arise in a paracrine fashion, as factors secreted by tumour (senescent) cells can induce senescence in the stroma or render infiltrating immune cells senescent. For example, implanting different tumour cells grafts (breast, pancreas, endometria, lung) in a p16^INK4a^ luciferase reporter mice results in luciferase activity arising in the tumour‐associated stroma, demonstrating the ability of tumours to induce senescence in their surroundings [41].

## Paradoxical roles of senescence in cancer

Senescence exerts multiple and sometimes opposing effects in tumorigenesis. The oncogenic activation events involved in cancer initiation trigger OIS in preneoplastic lesions and limit their progression. Consequently, mutations that disable senescence are needed for tumours to progress to more malignant stages. Most cancer therapies work, at least in part, by triggering senescence (TIS). But TIS in non‐cancerous cells has been linked to some of the side effects associated with chemotherapy [37]. And lingering senescent cells present in the tumour and TME contribute to sustain cancer development and progression [42].

### Antitumorigenic roles of senescence

Events that drive cancer initiation, such as activation of oncogenes (e.g., RAS or BRAF) or loss of tumour suppressors (e.g., PTEN), trigger OIS (in the case of PTEN loss also known as PTEN loss‐induced cellular senescence or PICS). OIS restricts tumorigenesis by imposing a stable cell cycle arrest in preneoplastic cells [32, 33, 34] that facilitates the subsequent elimination of these cells by the immune system, thus contributing to tumour clearance [43]. Surveillance by the immune system is an important part of the antitumour senescence response. Senescence immunosurveillance is initiated upon secretion of immunomodulatory cytokines by senescent cells as part of the SASP. For example, premalignant senescent hepatocytes are cleared *in vivo* through a CD4^+^ T cells response requiring monocytes and macrophages to actively eliminate the senescent cells [29]. This clearance is dependent of expression of IL1, CCL2 and other SASP factors [9]. The initiation of immune surveillance precedes full senescence: induction of p21 is sufficient to promote a secretome (termed PASP or p21‐activated secretory phenotype) that can attract macrophages [44]. The SASP, produced by hepatocytes [44] or senescent stellate cells [45], induces M1 polarisation in macrophages, favouring clearance of senescent cells. On the contrary, p53‐deficient stellate cells promote an M2 polarisation, creating a pro‐tumorigenic microenvironment which stimulates the proliferation of cancer cells [45]. Mouse liver carcinomas undergoing senescence recruit NK cells in a way that promotes the recruitment of NK cells, and the elimination of senescent tumour cells through a NKG2D‐dependent response [43, 46]. In summary, senescence limits cancer progression by triggering arrest of cancerous cells and activating different components of the immune system.

### Disabling senescence is needed for tumour progression and resistance to therapy

Although the senescence cell‐cycle arrest is considered as irreversible, it is believed that cells that have undergone senescence could re‐entry cell cycle [47]. This might explain how tumours progress or become resistant to anticancer therapies. There is evidence of senescent cells escaping from replicative senescence [47] and OIS [48]. Gorgoulis' group recently defined the concept of the escape from oncogene‐induced senescence (EOIS) helped by cellular models where deregulation of oncogenic signalling results in EOIS [48]. Upon CDC6 expression in epithelial cells, an early chromosomal reorganisation (inversion of chromosome 3) induces the circadian transcription factor Basic Helix–Loop–Helix Family Member E40 BHLHE40 activation (circadian clock machinery regulation). This is associated with senescent cells' re‐entry in cell cycle, likewise demonstrating a link between genomic instability and senescence escape [48].

Cancer cells can also escape TIS. MCF‐7 breast cancer cell clones treated with therapeutic doses of doxorubicin, after a burst of senescence induction, can recover and regain their proliferative ability that is associated with CDC2 overexpression [49]. H1229 non‐small cell lung cancer cells exposed to a variety of chemotherapeutic agents can also escape from TIS by overexpressing CDC2/CDK1, what is a very rare event [50]. Induction of senescence has been also shown to induce reprogramming. Interestingly lymphoma cells that have undergone senescence show an upregulation of reprogramming and display enhanced aggressiveness [51]. A recent study on primary acute myeloid leukaemia (AML) patients' cells revealed that even if chemotherapy induces a senescence‐like phenotype, this phenotype is transient and contributes to cancer relapse by promoting aggressiveness and stem cell potential of tumour cells [52]. Cancer cells that escape from TIS are often polyploid [53] and express markers of stemness and aggressiveness [54]. Senescent tumour cells can represent a form of “cell dormancy”, and thus also contributes to disease recurrence [55].

### Pro‐tumorigenic roles of senescence

While senescence can act as an intrinsic or evoked antitumour response, in the long term, the persistence of senescent cells has detrimental effects on tumorigenesis, promoting tumour progression, dissemination and recurrence [37, 56].

Tumour cell senescence can lead to the emergence of more aggressive variants. Particularly, chemotherapy‐induced senescence can reprogram cancer cells which acquired stemness properties, thus enhancing their aggressiveness, resistance to therapy and relapse [51]. TIS can also promote cancer relapse by preventing the induction of apoptosis by chemotherapeutics. In the context of wild‐type p53, doxorubicin failed to induce apoptosis of mammary tumours in mouse model, while p53 mutant cells did not undergo cell cycle arrest and were killed due to mitotic abnormalities [56]. This indicates that p53 status is an important factor influencing cancer therapy, in part by affecting senescence induction.

Senescent cells communicate with adjacent cells via ligands/receptors interactions to modulate their state and their microenvironment [6, 7]. Senescent cells can promote the proliferation of adjacent tumour cells in a paracrine manner via the SASP [57]. For example, in a model of paediatric craniopharyngioma, oncogene activation in pituitary stem cells leads to senescence and the SASP drives tumorigenesis in a paracrine manner [58]. The SASP can also reinforce senescence or cause paracrine senescence [9, 19]. During therapy‐induced senescence (TIS), especially upon radiotherapy, there is a bystander signalling mediated by inflammatory mediators and gap junctions that can be related to paracrine senescence [59]. Whether the reinforced senescent phenotype will allow a better immune clearance of the cells or in the contrary, confer a higher pro‐tumorigenic environment could depend on the context. The SASP of senescent cells lingering in the tumour microenvironment contributes to create a chronic inflammatory environment [60]. The SASP of senescent fibroblasts can promote tumour growth and dissemination, in part through the induction of epithelial‐mesenchymal transition (EMT) in adjacent cells [61]. The SASP also influences immune responses beyond the positive effects of senescence immunosurveillance. While SASP production stimulates the clearance of senescent cells by the immune system [9, 29], when senescence is bypassed or if the senescent cells are not fully eliminated, senescent cancer cells can reshape the immune microenvironment promoting immune escape. For example, in the context of liver cancer, the SASP can recruit CCR2+ immature myeloid cells (iMCs) that inhibit NK cell action, promoting cancer progression [62]. Similarly, senescence observed in PTEN‐null prostate cancer results in immature myeloid cell infiltration that antagonises senescence, promoting tumour progression [63].

A senescent stroma plays detrimental role in cancer. Co‐injection of senescent fibroblasts with skin, breast or prostate cancer cells favours the aggressiveness of the generated tumours [39, 64, 65]. The tumour‐promoting effect of senescent fibroblasts is likely to be mediated by the secretion of SASP factors that can foster a growth‐stimulating and/or pro‐angiogenic microenvironment [66]. Senescent endothelial cells are also present in the tumour microenvironment and secrete factors that influence the tumour behaviour [67]. For example, SASP factors like CXCL11 secreted by senescent endothelial cells contribute to the cancer aggressiveness of breast cancer [68].

Fostering immunosuppression is another way by which senescent cells can promote tumour progression. For example, in a model of aged skin, senescent stromal cells recruit suppressive myeloid cells and secrete SASP factors like IL‐6 to reinforce immunosuppression. The same observation was made in cancer patients where senescent stromal cells are adjacent to immune cells, thus creating a tumour permissive microenvironment [39]. Other strategy used by senescent cells to escape immune surveillance is the matrix metalloproteinase (MMP)‐mediated shedding of NKG2D ligands [69]. Elimination of senescent cells by NK cells involves the recognition of NKG2D ligands at their cell surface. While the expression of NKG2D ligands is induced during senescence, senescent cells also secrete MMPs, that cleave NKG2D ligands, avoiding NK surveillance.

## Cancer therapy and senescence‐inducing drugs

Many cancer therapeutics induce senescence. Examples include drugs that trigger DNA damage, i.e., genotoxic chemotherapy or radiotherapy (Table 1). Various targeted therapies, such as CDK4/6 inhibitors, can also induce senescence without being genotoxic (Table 2). Some of these senescence‐inducing drugs can cause senescence not only in the tumour itself but also in the TME. Thus, induction of senescence by cancer therapies plays key role in cancer treatment but can also cause unwanted side effects associated with lingering senescent cells.

**Table 1:** Senescence inducers: conventional chemotherapy and radiotherapy.

Class	Drug	Cancer	Model
Alkylating agents	Cisplatin	Fibrosarcoma	HT1080 [70]
		Melanoma	A375, B16F10 [71]
		Nasopharyngeal carcinoma	CNE1 [72]
	Cyclophosphamide	B‐cell lymphoma	Eμ‐Myc;ectopic Bcl2 mouse model [73]
	Temozolomide	Glioma	U87MG [74, 75]; GaMG [74]; U87, LN229 [76]; LN229 [77]
		Melanoma	MM200, IgR3, SK‐MEL‐28, Mel‐FH [78]
Topoisomerase inhibitors	Doxorubicin	B‐cell lymphoma	Eμ‐*Nras* ^G12V^;ectopic *Bcl2* mouse model [34]
		Breast cancer	MCF‐7 [70, 79, 80]; Reporter Mouse model p16‐3MR‐MMTV‐PyMT grafts [37]; patients [79]
		Cervical carcinoma	HeLa [70]
		Colon cancer	HCT‐116, SW480 [70]; LS174T, HCA‐7 [79]
		Fibrosarcoma	HT1080 [70]
		Glioma	U251 [70]
		HCC	HepG2 [70]
		Larynx carcinoma	Hep‐2 [70]
		Lung cancer	NCI‐H460, A549 [81]
		Osteosarcoma	Saos2 [70]
		Ovarian cancer	A2780 [70, 79]
		Prostate cancer	LNCaP [70, 82]; DU145 [82]
	Daunorubicin	T‐cell lymphoma	Jurkat T cells [83]
	Etoposide	Fibrosarcoma	HT1080 [70]
		HCC	HepG2, U2OS [84]
	Irinotecan	Colorectal cancer	HCT‐116 [85, 86, 87]; LS174T [86, 87]
Microtubule inhibitors	Docetaxel	Prostate cancer	LNCaP, DU145 [82]; *Pten* ^pc−/−^ mouse model [88]
	Paclitaxel	Breast cancer	MCF‐7 [89, 90]; MDA‐MB‐231 [90]
		Colorectal cancer	HCT‐116 [90]
		Melanoma	A549 [90]
		Neuroblastoma	SH‐SY5Y [90]
Antimetabolites	Gemcitabine	Pancreatic carcinoma	Panc1 [91, 92]; Miapaca‐2 [92]
	Methotrexate	Breast cancer	MCF‐7 [93]
Cytotoxic antibiotic	Bleomycin	Lung cancer	A549 [94, 95]
Radiotherapy		Breast cancer	MCF‐7 [96]
		Glioma	U87 [97]

**Table 2:** Senescence inducers: targeted therapies.

Class	Drug	Cancer	Model
CDK4/6 inhibitors	Palbociclib	Breast cancer	V720 [98]; MCF‐7, T47D [99]
		Gastric cancer	AGS, MKN‐45 [100]
		Glioblastoma	DBTRG‐05MG, LN229, U87MG [101]
		HNSCC	CAL27, HN31, PCI15B [102]
		HCC	Huh7, SK‐Hep1 [103]
		Liposarcoma	U2OS [104]; LS8107 [105]
		Lung cancer	A549, H2030, H460 [106]; *LSL‐Kra*s^G12V^;*RERT* ^+/ERT^ mouse model [107]
		Melanoma	SK‐MEL‐103, SK‐MEL‐103 xenografts [108]; MEL‐10 [109]; SKMEL2 [98]
		OSCC	SAS, OECM1 [110]
		Osteosarcoma	U2OS [98]
	Abemaciclib	Breast cancer	MD‐MB‐453 [111, 112, 113]; BT474 [113]; MCF‐7 [114]; MMTV‐rtTA/tetO‐HER2 mouse model [113]
	Ribociclib	Ewing sarcoma	SKNEP‐1 [115]
		Neuroblastoma	BE2C, IMR5, EBC1 [116]
		Ovarian cancer	Hey1 [117]
Epigenetic modulators	5‐azacytidine	HCC	HepG2 [118, 119]; Huh‐7 [119]
		Prostate cancer	LNCaP, C4‐2B [82]
	5‐aza‐2′‐deoxycytidine/decitabine	CML	K‐562, MEG‐01, KBM‐5 [120]
		Lung mesothelioma	H28 [121]
		Osteosarcoma	U2OS [122]
HDACi	SAHA/vorinostat	AML	MOLM‐7, HL‐60, JURK‐MK1 [123]
		Colorectal cancer	HCT‐116 [124]
		T‐cell lymphoma	MyLa, MJ [125]
	Sirtinol	Breast cancer	MCF‐7 [126, 127]
		CML	K562 [128]
		Lung cancer	H1299 [126]
AURK inhibitors	Alisertib	Colorectal carcinoma	HCT‐116, HCT‐116 xenografts [129]
		Glioblastoma	GB169 [130]
		Lung cancer	A549 [131]
		Pancreatic cancer	AsPC‐1, BxPC‐3, MIA PaCa‐2, PANC‐1 [132]
	Barasertib	Lung cancer	A549 [131]
		Melanoma	A375 [133]
	Danusertib	Glioblastoma	GBM2, G166 [134]
	Tozasertib	Lung cancer	A549 [131]
PLK inhibitors	BI‐2536	Colorectal carcinoma	HCT‐116 [135, 136]; SW480, HCT‐116 xenografts [135]
		Lung cancer	A549 [135]
	BI‐6727/volasertib	Colorectal carcinoma	HCT‐116 [136]
		Lung cancer	A549 [137]
	CFI‐400945	HCC	MHCC97L [138]
		Ovarian cancer	OCC1, ES2 [139]
		Prostate cancer	22Rv1, DU145, PC‐3, LNCaP, C4‐2 [140]
PARP inhibitors	Olaparib	AML	KASUMI and NB4‐LR2 [141]
		Breast cancer	MDA‐MB‐231, OV4453, OV1946 and MDA‐MB‐231 xenografts [142]
		Ovarian cancer	OV1369(R2), OV90, OV4453, OV1946 [142]; SKVO3, A2780, OVCAR‐3 [143]
		Prostate cancer	LNCaP, C4‐2B [144]
	Rucaparib	Prostate cancer	LNCaP, C4‐2B, PC‐3 [145]
	Niraparib/Talazoparib	Ovarian cancer	OV1369(R2) [142]
	ABT‐888/veliparib	Breast cancer	MCF‐7, MCF‐7 xenografts [146]
Cdc7 inhibitor	XL413	HCC	Hep3B, Huh7, MHCC97H, PLC/PRF/5 p53 mutant cells [147]
Hormone therapy	Tamoxifen	Breast cancer	MCF‐7 [148, 149]
	ADT	Prostate cancer	LNCaP [150, 151]; LAPC4; LuCaP [151]; Patient biopsies [151]
	Bicalutamide	Prostate cancer	LNCaP, LAPC4 [152]
Immunotherapy	Rituximab	B cell lymphoma	EHEB, RC‐48, SD1 [153]
	Trastuzumab & pertuzumab	Breast cancer	SK‐BR‐3 [154]
VEGF/VEGFR inhibitors	Bevacizumab	Colorectal cancer	MIP101, RKO, SW620, SW480 [155]
	AZD‐2171	Colorectal cancer	HCT‐116 [156]
MEK inhibitor	Trametinib	Melanoma	A375, D04 [157]; DMBC11, DMBC12, DMBC21, DMBC28, DMBC17 [158]
		Lung cancer	A549, H2030, H460 [106]
B‐Raf inhibitor	Vemurafenib	Melanoma	DMBC11, DMBC12, DMBC21, DMBC28, DMBC17 [158]; M14, M19‐Mel, Malme 3 M, SK‐MEL‐28, UACC‐62, Mel2a, FM88 [159]
SKP2 inhibitor		Lung cancer	A549, H1299 [160]
		Prostate cancer	PC‐3 [161, 162]

### Chemotherapies

Cytotoxic chemotherapy includes a wide range of drugs that inhibit mitosis or induce DNA damage in cancer cells. Pioneering studies showed that besides killing them, agents such as cisplatin [72] and doxorubicin [70] can also induce senescence in cancer cells. Lower drug dose and chronic administration often result in senescence rather than apoptosis and cell death [163].

Alkylating agents, mainly represented by cisplatin and derivates, alkylate and bind covalently the DNA to form DNA crosslinks. These structures are susceptible to breakage during DNA replication and may lead to the activation of DDR. In the same way, topoisomerase inhibitors like doxorubicin or etoposide affect the ability of the DNA to replicate, by preventing the repair of DNA breaks required to reduce the tension of the unwound DNA strand during the replication. Thus, DNA damage triggers activation of p53/p21 [164] or p16 [73] and causes the subsequent proliferation arrest and senescence of cancer cells. Treatment with different chemotherapeutic agents also results in SASP induction.

So‐called antimicrotubule agents, such as the taxanes paclitaxel and docetaxel, and vinca‐alkaloids, are a class of cancer drugs that interfere with normal mitosis by causing microtubule dysfunction. Paclitaxel has been described to induce senescence of breast cancer [89], melanoma and neuroblastoma cells [90]. Docetaxel has been shown to induce senescence in different models of prostate cancer, where it is used therapeutically [82, 88]. Antimetabolites, that impair the incorporation of purines and pyrimidines to the DNA during the S phase, also induce senescence. Gemcitabine and methotrexate have been described as senescence inducers, in a p21‐dependent fashion [91, 92]. Methotrexate treatment seems to induce a p53‐dependent senescence without DNA damage [93] that could be linked to reduced DNA synthesis [165]. Finally, bleomycin, a cytotoxic antibiotic used to treat many cancer types, induces a p21‐dependent senescence response [94, 95].

### Radiotherapy

Radiotherapy is used to treat many cancer types. Like chemotherapies, irradiation not only causes cell death but can also result in senescence. Either γ‐irradiation [70] or exposure to x‐ray [166] triggers a senescent response. Induction of the senescent phenotype is dependent on the p53 function and induction of DDR in telomeres but independent of telomere shortening [167]. One of the advantages of radiotherapy when compared with cytotoxic chemotherapies is their local administration, which reduces side effects. Despite that, irradiation of surrounding healthy tissue can cause side effects such as radiation‐induced lung fibrosis (RILF), a severe side effect of radiotherapy observed in lung cancer patients [168]. Senescence of cells in the TME (macrophages, pneumocytes and fibroblasts) is known to be part of this complication [169, 170, 171]. Interestingly, FLASH irradiation (consisting in the delivery of large doses of radiation in a fraction of second) minimises the DNA damage of normal lung cells *in vitro* and of irradiated mice lung *in vivo* compared to conventional radiotherapy, thus preserving normal lung from radio‐induced senescence and associated lung fibrosis [172].

### CDK4/6 inhibitors

Cyclin‐dependent kinases 4 and 6 (CDK4/6) are responsible for the cell cycle transition from G1 to S phase by phosphorylating RB family proteins. Cancer cells often present a hyperactivated CDK4/6 to sustain their proliferation. Therefore, CDK4/6 inhibitors are a promising new cancer therapy [173]. The first CDK4/6 inhibitor, palbociclib, was described in 2004 and later approved by the FDA as a treatment for hormone receptor (HR) + HER2‐ breast cancer [174]. Two other CDK4/6 inhibitors abemaciclib [175] and ribociclib [176] are currently used in the clinic, while others are under evaluation [177]. CDK4/6 activity is needed for cell cycle progression and is often upregulated in tumours, frequently in absence of genetic alterations but due to the upregulation of mitogenic signalling [111]. CDK4/6 inhibitors inhibit E2F transcriptional activity leading to an RB‐dependent cell proliferative arrest. While CDK4/6 inhibitors can induce quiescence, they also can trigger senescence in various cancer cells [178, 179]. Acquired resistance to CDK4/6 inhibitors occurs in highly responsive HR‐positive breast cancer. Loss of *RB* and overexpression of cyclin E are associated with palbociclib resistance [180, 181, 182]. Pandey et al. also demonstrated the overexpression of cyclin E in palbociclib‐resistant cells. Cyclin E‐CDK2 interaction could be targeted by inhibition of CDK2, and combined inhibition of CDK2‐CDK4/6 synergistically overcomes CDK4/6 resistance and enhances senescence, highlighting the therapeutic relevance of senescence [183].

### AURK/PLK inhibitors

Aurora kinases (AURK) are essential serine/threonine kinases that control spindle formation and mitotic progression [184]. Aurora kinases are overexpressed in a broad range of human tumours, including gastrointestinal, breast, ovarian and pancreatic cancer [185]. The expression of AURK is linked to genomic instability and cancer, and due to their role as cell cycle regulators, their inhibition is of great interest for cancer therapy [186]. Inhibitors of Aurora kinases have been proven effective as cancer treatments [187]. Interestingly, the Aurora A inhibitor alisertib, Aurora B inhibitor barasertib and the pan Aurora A/B/C inhibitors danusertib and tozasertib are potent inducers of senescence in cancer cells [129, 131].

Polo‐like kinases (PLK) are another family of serine/threonine kinases essential for cell mitosis [188]. PLK1 is overexpressed in a wide range of tumours [189]. Studies demonstrated the therapeutic value of PLK1 inhibition, particularly in endocrine‐resistant breast cancer, as overexpression of PLK1 is observed in the patient with cancer relapse [190]. The PLK1 inhibitor BI2536 enhances the effect of paclitaxel on MCF‐7 and T‐47D breast cancer cells, by inducing their apoptosis, whereas it prevents tamoxifen‐induced senescence [191]. On the contrary, PLK1 inhibition by MLN0905 promotes senescence in a variety of cancer cells [135]. Interestingly CFI‐400945, an inhibitor of the PLK family member PLK4, induces senescence in cancer cells through cytosolic DNA accumulation and activation of the cGAS/STING pathway [138, 139].

### Epigenetic modulators

Several drugs that target epigenetic regulators cause senescence. For example, 5‐azacytidine and its deoxy derivate 5‐aza‐2′‐deoxycytidine (decitabine) are analogues of the nucleoside cytidine and used in cancer therapy as inhibitors of DNA methyltransferase [192]. While developed as cytostatic drugs, particularly in haematological cancers, the response in cancer cells was further investigated to demonstrate that senescence induction also contributes to their antiproliferative effects [82, 121]. The mechanism of senescent induction and its ability to induce senescence or apoptosis depends on context. 5‐aza‐2′‐deoxycytidine but not 5‐azacytidine induces profound DNA damage leading to senescence. On the contrary, 5‐azacytidine, known to block RNA synthesis, decreases p53 protein accumulation and leads to the apoptosis of HepG2 cells [118]. Histone deacetylase (HDAC) inhibitors such as vorinostat (suberoyanilide hydroxamic acid, SAHA) are used for the treatment of different cancers such as cutaneous T‐cell lymphomas. HDAC inhibitors cause senescence in different cancer types [124, 125]. The SIRT1 inhibitor sirtinol also causes senescence, although the precise mechanism responsible is unclear [126, 127, 128]. More recently, an inhibitor of the histone lysine acetyltransferases (KATs) KAT6A and KAT6B, has been shown to induce *INK4/ARF*‐dependent senescence in cancer cell lines, explaining their ability to hinder lymphoma progression [193].

### PARP inhibitors

Poly(ADP‐ribose) polymerase (PARP) is involved in the detection of single‐strand breaks (SSBs) and plays important roles in regulating DNA repair and genomic stability. BRCA1 or BRCA2 mutant tumours are sensitive to PARP inhibitors (PARPi) due to their defects on DNA repair [194]. Other tumours with DNA repair defects also display synthetic lethality with PARPi. In other cases, treatment with PARPi causes senescence. For example, treatment of MCF‐7 breast cancer cells with veliparib increases the number of DNA damage foci observed in response to radiation, inducing senescence [146]. This suggested that DNA damage sensitises the cancer cells to the senescence induced by PARPi. Indeed, KASUMI and NB4‐LR2 AML cells, bearing compromised DDR genetic alterations, are sensitive to olaparib‐induced senescence, in contrary to THP‐1 cells [141]. Similar responses have been observed in ovarian [143] and prostate cancers [144, 195]. In ovarian cancer, PARPi induces a reversible senescent‐like phenotype that caused a BCL‐xL‐mediated resistance to apoptosis [142].

### Other senescence‐inducing drugs

A number of other drugs and antibodies can induce senescence in cancer cells. In hormone‐dependant cancers, hormone‐receptor antagonists like tamoxifen in breast cancer or bicalutamide in prostate cancer not only inhibit tumour growth but can also induce senescence [148, 149, 150, 151, 152]. In breast cancer, targeting HER2/neu with trastuzumab or pertuzumab also causes senescence [154]. In melanoma, BRAF‐activating mutations are approximatively present in 50% of all tumours [196]. MAPK2K1 and MAPK2K1 encoding MEK1/2 are frequently mutated in melanoma, overall leading to the hyperactivation of MAP kinase pathway [197]. The development of BRAF and MEK inhibitors (respectively vemurafenib and trametinib, among others), shows great antitumorigenic effect in patients while also inducing senescence and contributing to radiosensitise BRAF‐mutated melanoma cells but also to explain the absence of complete response to vemurafenib [157, 158, 159]. VEGFR2 inhibition that results in AKT downregulation also induces p21 and triggers senescence in colorectal cancer cells [156]. Similar results were obtained with the VEGF inhibitor bevacizumab [155]. The SKP2 E3‐ubiquitin ligase is described as an oncogene in many cancers. SKP2 inhibitors exhibit antitumour activities and leads to a p53‐independent, p21/p27‐dependent induction of senescence in lung and prostate cancers [160, 161, 162]. Finally, rituximab anti‐CD20 immunotherapy exerts antilymphoma activity but also displays a pro‐senescence activity towards B‐cell lymphoma, thus sensitising lymphoma cells to conventional chemotherapies [153].

## Senolytics and other senotherapeutics

Senescence has both detrimental and beneficial roles in disease (and cancer) progression. That paradoxical behaviour made that for many years it was not obvious what the best way to exploit senescence therapeutically was. That changed with the development of mouse models (such as the INK‐ATTAC mice) allowing for the selective ablation of senescent cells (senolysis). In a series of seminal studies, it was shown that eliminating senescent cells improved lifespan, healthspan and ameliorates many age‐related diseases [198, 199, 200]. That prompted the search for drugs (so‐called senolytics) that selectively kill senescent cells and have the potential to be used against a myriad of age‐related diseases, including cancer (Table 3).

**Table 3:** Uses of senolytic drugs in preclinical models of cancer.

Class	Drug	Cancer	Senescence induction	Model
BH3 mimetics	ABT‐263/navitoclax	Breast cancer	Irradiation, paclitaxel	4226, Cal51 [201]
			Doxorubicin	MDA‐MB‐231, MDA‐MB‐231 xenograft [202]
		Colorectal cancer	Etoposide	HCT‐116 [203]
		Glioblastoma	Irradiation	GBM39, GBM76, GBM6, GBM123 [204]
			Temozolomide	GBM39, GBM76 [204]
		Lung cancer	Etoposide, irradiation	A549, A549 xenograft [202]
		Breast, lung cancer, osteosarcoma	Doxorubicin	4226, SKBR7, Cal51, U2OS, A549 [201]
		Lung, breast, colorectal, hepatocellular cancers	Alisertib or etoposide	A549, MDA‐MB‐231, SUM159, RKO, PC9, MCF‐7, Huh7, Hep3B, H358, T47D, HepG2 [203].
		Ovarian and breast cancers	Olaparib	OV1946, OV4453, MDA‐MB‐231 [142]
	ABT‐199/venetoclax	Breast cancer	Palbociclib	MCF‐7, patient tumour organoid, PDX [205]
		Glioblastoma	Temozolomide	LN‐229 [206]
		Sarcoma	Irradiation	STS117, STS93, STS109 [207]
	ABT‐263/navitoclax + S63845 MCL‐1 inhibitor	Breast cancer	Doxorubicin	HCC712, MDA‐MB‐175, MCF‐7, 747D [201]
			Irradiation, paclitaxel	MDA‐MB‐175 [201]
	S63845 MCL‐1 inhibitor	Prostate cancer	Docetaxel	PC3 Luc‐ShTimp1 xenografts [208]
Cardiac glycosides	Ouabain	Craniopharyngioma	Oncogenic β‐Catenin	Hesx1^Cre/+^;Ctnnb1^lox(ex3)/+^ mouse model [209]
		Colorectal cancer	Doxorubicin	HCT‐116 [209]
		Preneoplastic hepatocytes	NRAS^G12V^ expression	NRAS^G12V^ hydrodynamic injection in mice [209]
		Hepatocellular carcinoma, lung cancer	Etoposide, Barasertib, Alisertib, Tozasertib, Palbociclib	SK‐Hep1 and A549 [209] [210]
		Lung cancer	Etoposide, doxorubicin	A549 [210]
			Bleomycin, Gemcitabine, Doxorubicin, Etoposide, Palbociclib	A549 [211]
		Melanoma	Palbociclib	SK‐MEL‐103 [211]
	Digitoxin	Hepatocellular carcinoma, lung cancer	Etoposide, Barasertib, Alisertib, Tozasertib, Palbociclib	SK‐Hep1 and A549 [209]
	Digoxin	Breast cancer	Doxorubicin	PDX [211]
		Lung cancer	Bleomycin	A549 [211]
		Lung cancer	Gemcitabine	A549 xenografts [211]
		Melanoma	Palbociclib	SK‐MEL‐103 [211]
	Proscillaridin A	Lung cancer	Bleomycin	A549 [211]
Galacto‐conjugates	Gal‐NP(Nav)	Breast cancer	Palbociclib	Xenograft [212]
	Nav‐Gal	Lung cancer	Cisplatin	A549 + xenograft [213]
	Galactose‐modified duocarmycin	Craniopharyngioma	Oncogenic β‐Catenin	Hesx1^Cre/+;^Ctnnb1^lox(ex3)/+^ [214]
BET inhibitor	ARV825	Colorectal carcinoma	Doxorubicin	HCT‐116 [215]
		Hepatocellular carcinoma		Obesity‐induced HCC mouse model [215]
HDAC inhibitor	Panobinostat	HNSCC	Taxol, cisplatin	FaDu, UMSCC47 [216]
		Lung cancer	Taxol, cisplatin	A549, H460 [216]
FOXO4 peptidomimetics		Melanoma	Doxorubicin	A375, A375 grafts [217]
mTOR inhibitors	AZD8055 and AZD2014	Hepatocellular carcinoma	XL413 (CDC7 inhibitor)	Huh7, Hep3B; Huh7 and MHCC97H xenografts; Myc^OE^;Trp53^KO^‐HCC bearing mice [147]

### BH3 mimetics

Many stresses (such as irradiation or chemotherapy) can either induce senescence or trigger cell death. Consequently, for cells to undergo senescence they not only need to exit the cell cycle but also inhibit apoptosis. One mechanism by which senescent cells prevent cell death is by upregulating anti‐apoptotic BCL‐2 family proteins [218]. BH3 mimetics are a class of drugs that inhibit BCL2 family inhibitors. ABT‐737 was one of the first BH3 mimetic drugs developed and has selectivity towards BCL‐2, BCL‐xL and BCL‐W [219]. Other drugs, such as ABT‐263/navitoclax (orally bioavailable) [220] and ABT‐199/venetoclax (a selective BCL‐2 inhibitor) were derived from it [221].

Three pioneering studies showed that ABT‐263 and ABT‐737 can selectively eliminate senescent cells [222, 223, 224]. Upregulation of anti‐apoptotic BCL‐2 family proteins BCL‐2, BCL‐xL and BCL‐W during senescence was suggested as the basis for this sensitivity to BH3 mimetics [218]. Besides eliminating a wide‐range of mouse and human senescent cells, navitoclax (ABT‐263) can rejuvenate the haematopoietic system of irradiated mice [223] and ABT‐737 selectively eliminated senescent cells in lung and skin [218]. Importantly, in most cell types ABT‐263/navitoclax (a BCL‐2 and BCL‐xL inhibitor) but not ABT‐199/venetoclax (a selective inhibitor for BCL‐2) is senolytic, although there are exceptions. Navitoclax causes severe thrombocytopenia, what has limited its clinical use and encouraged the development of more selective BCL‐2 inhibitors, such as ABT‐199/venetoclax [225].

Nevertheless, not all senescent cells are sensitive to ABT‐263. For example, a recent study of senescent cancer cells showed that LoVo cells undergoing senescence in response to alisertib or etoposide treatment are insensitive to ABT‐263 treatment [203]. Similarly, senescent prostate cancer cells treated with antiandrogens such as enzalutamide are resistant to ABT‐263 treatment [226]. Multiple factors are likely responsible of the different sensitivity to BH3 mimetics. Context‐dependent induction of different antiapoptotic BCL‐2 family proteins, such as MCL‐1 might be an explanation. Recently, scRNA sequencing of prostate cancer cells from *Pten* ^pc−/−^ and *Pten* ^pc−/−^;*Timp1* ^−/−^ mouse models showed *Mcl‐1* induction upon senescence. Consistently, the MCL‐1 inhibitors S63845, UMI77 and AZD5991 efficiently eliminated those senescent prostate cancer cells [208].

### Quercetin, fisetin and other flavonoids

Senescent cells activate anti‐apoptotic and pro‐survival pathways. Quercetin was identified as a senolytic drug chosen from selected candidates regulating anti‐apoptotic and pro‐survival pathways induced during senescence [227]. In that study, it was shown that the combination of the tyrosine kinase inhibitor (TKI) dasatinib and quercetin (D + Q) was promising in the clearance of senescent cells. Interestingly, D + Q effectively cleared senescent cells of old, progeroid *Ercc* ^1−/Δ^ mice and irradiated mice to increase their cardiovascular function, healthspan and physical endurance [227]. Following the discovery of quercetin as a senolytic, a panel of flavonoids was screened to test their capacity to kill senescent mice and human fibroblasts [228]. Among the flavonoids tested, fisetin demonstrated senolytic activity on *Ercc1* ^−/−^ mouse embryonic fibroblasts (MEFs) and etoposide‐treated IMR‐90 fibroblasts and reduced senescent cell burden of *p16* ^+/Luc^;*Ercc1* ^−/Δ^ progeroid mice, aged mice, and in human explants. Fisetin eliminates senescent human umbilical vein endothelial cells (HUVECs), but not senescent IMR‐90 cells or primary human adipocytes [229]. Procyanidin C1, another flavonoid, has also been recently shown to display senolytic properties [230].

While flavonoids are commonly consumed in human diet, none of these compounds are currently approved for medical use, despite numerous publications on beneficial effects to treat cancer or cardiovascular disease. Recently, first‐in‐human studies have suggested the efficacy of the senolytic combination dasatinib + quercetin in the improvement of the condition of patients with pulmonary fibrosis and chronic kidney disease [231, 232]. Several clinical trials are currently in progress to evaluate the senolytic activity of quercetin, fisetin (NCT05276895; NCT04815902; NCT04770064) and dasatinib + quercetin (NCT04063124; NCT04685590, NCT02848131; NCT04313634) in patients.

### FOXO4 peptidomimetics

FOXO4 is upregulated in senescent cells and contributes to maintain their viability. FOXO4 resides in PML bodies and colocalises with DNA segments with chromatin alterations reinforcing senescence (DNA‐SCARS) where it interacts and sequesters p53 in the nucleus, to prevent apoptosis. Thus, FOXO4‐DRI peptides [D‐Retro Inverso (DRI)‐isoform] compete with endogenous FOXO4, releasing p53 and causing apoptosis of senescent cells [233]. This strategy was successfully validated on irradiated IMR‐90 fibroblasts and eliminates senescent cells in premature ageing mouse models and mice treated with doxorubicin [233]. More recently, other FOXO4 peptidomimetics have been developed [217]. These new peptides showed 3–7 times more effective senolytic activity on doxorubicin‐treated A375 melanoma cells and orthotopic grafts.

### Galacto‐conjugated senolytics

Senescent cells display an increase in lysosomal mass that results in higher activity of lysosomal β‐galactosidase [31]. As a result, SA‐β‐Gal activity is one of the most frequently used markers for senescence. Several strategies have taken advantage of the higher β‐galactosidase activity of senescent cells to design drugs with enhanced selectivity towards senescent cells.

This rationale was first tested by designing galacto‐oligosaccharide‐capped nanoparticles, which preferentially released their cargo on senescent cells [234]. This strategy could be combined with imaging agents to identify senescent cells. Encapsulation limited the cytotoxic effects of doxorubicin and transformed it into a senolytic drug. In addition, nano‐encapsulated navitoclax Gal‐NP(Nav) inhibited the tumour growth and the emergence of metastases, while limiting the whole‐body toxicity of navitoclax [108].

Galactose conjugation was taken a step further by using galactose‐derived pro‐drugs rather than encapsulating drugs in galacto‐oligosaccharide‐capped nanoparticles. A galactose derivative prodrug of navitoclax (Nav‐Gal) showed a higher specificity toward cisplatin‐induced senescent A549 cancer cells compared to unconjugated navitoclax. *In vivo*, the concomitant treatment with cisplatin and Nav‐Gal cleared the senescent cancer cells and reduced the growth of cancer xenografts, sparing platelets and not causing thrombocytopenia [213]. Other studies converted cytotoxic drugs into senolytics by generating galactose‐conjugated pro‐drugs. For example, galactose prodrug derivatives of the alkylating antibiotic duocarmycin displayed senolytic activity both in culture and *in vivo* [214]. Similarly, SSK1, a galactose prodrug derived from gemcitabine targeted senescent cells, attenuated inflammation and restore the physical activity of old mice [235].

### Cardiac glycosides

Unbiased phenotypic screens have also served to identify senolytic drugs. Taking these approaches, two groups independently identified the senolytic potential of cardiac glycosides [209, 211]. Cardiac glycosides (CGs), such as ouabain, digoxin and digitoxin, are natural compounds used in cardiology as inhibitors of Na^+^/K^+^‐ATPase. Interestingly, there is an increase in the concentration of intracellular cations in senescent cells, and treatment with CGs decrease K^+^ levels more prominently in senescent cells, what eventually cause induction of the pro‐apoptotic NOXA gene. Thus, the senolytic activity of cardiac glycosides might be explained by inhibition of the Na^+^/K^+^‐ATPase and depolarization of the plasma membrane. *In vivo*, CGs were able to eliminate different senescent cells including preneoplastic hepatocytes and naturally arising senescent cells in old mice [209]. Moreover, CGs synergise with gemcitabine or doxorubicin to eliminate cancer xenografts [211]. Interestingly, another inhibitor of ATPase pumps, curcumin, and derivatives such as o‐vanillin and EF24 also displays senolytic activity [209, 236, 237].

### Other small compounds with senolytic activity

Drug screens have served to identify other drugs with senolytic properties. HSP90 chaperone inhibitors such as geldanamycin, and its derivates 17‐AAG (tanespimycin) and 17‐DMAG (alvespimycin) downregulate the pro‐survival PI3K/AKT pathway, which is associated with a decreased number of senescent cells and an increased in healthspan of Ercc1^−/Δ^ progeroid mice [238].

Another drug screen identified the BET (Bromodomain and Extraterminal domain) family protein degrader (BETd) as a senolytic drug and validated the ARV825 compound as the BET inhibitor with the strongest senolytic activity [215]. ARV825 is a PROTAC (proteolysis targeting chimera), consisting in two active domains, one able to engage an E3 ligase to allow the proteolytic degradation of the chosen protein targeting by the second active domain. This PROTAC eliminates senescent cells in a model of obesity‐induced hepatocellular carcinoma (HCC), restraining tumour development. Other drugs, such as the HDAC inhibitor panobinostat have been shown to have senolytic activity. In A549 non‐small cell lung cancer (NSCLC) and FaDu head and neck squamous cell carcinoma (HNSCC) cells, taxol and panobinostat cotreatment showed a synergistic killing of cancer cells [216]. This apoptosis of the cancer cells was attributed to the induction of senescence by taxol followed by panobinostat‐induced senolysis.

### Exploiting the immune system to eliminate senescent cells

Beside small compounds, senescent cells can be targeted taking advantage of the immune system. For example, antibodies against senescent‐specific antigens such as DPP4 can be used to target senescent cells by potentiating antibody‐dependent cell‐mediated cytotoxicity (ADCC) [239]. An antibody against B2M, an extracellular epitope identified as a membrane marker of senescence, conjugated to duocarmycin was able to kill doxorubicin‐induced senescent HCT‐116 colorectal cancer cells [240]. Recently, the therapeutic concept of chimeric antigen receptor (CAR) T cells was applied to senotherapy. The urokinase plasminogen activator receptor (uPAR) is a cell surface protein upregulated in senescent cells. The authors thus designed CAR T cells directed against uPAR that successfully eliminate senescent cells *in vitro* and *in vivo* upon TIS in *Kras* ^G12D^;*Trp53* ^−/−^ lung adenocarcinoma mouse model, what resulted in extended survival [241].

### Other senotherapies

Besides the elimination of senescent cells by senolytics, senotherapies include senomorphic drugs that modulate the SASP to ensure the immune‐mediated senescent cell clearance. For example, the combination of MEK and CDK4/6 inhibitors induces senescence in *Kras*‐driven models of lung cancer and pancreatic ductal adenocarcinoma (PDAC). In lung cancer, that is sufficient to promote NK‐mediated regression [106], while in PDAC, the SASP increases tumour vascularization, facilitating chemotherapy delivery and efficacy, and increasing the recruitment of CD8^+^ T immune cells, thus sensitising these “cold” tumours to immunotherapy [242]. These studies suggest that immune response against senescent cancer cells could be further stimulated by immune checkpoint inhibitors (ICI).

Alternatively, senomorphic drugs altering the SASP phenotype without killing the senescent cells could be used to prevent the adverse effects of the remaining senescent cells or favour senescence immunosurveillance. Senomorphic drugs include IκB kinase (IKK) and NF‐κB kinase inhibitors [243], JAK/STAT signalling pathway inhibitors [244] or mTOR inhibitors [12, 13]. Interestingly, the antidiabetic drug metformin improves the healthspan and lifespan in mice, notably via the AMP‐activated protein kinase (AMPK) activation resulting in decreased oxidative damage and chronic inflammation [245, 246]. While metformin has pleiotropic effects, it also inhibits SASP production by preventing NF‐kB activation [247, 248]. Rapamycin, a selective inhibitor of mTOR, also inhibits the proinflammatory SASP [12, 13]. Everolimus, a second‐generation rapamycin derivate, is currently approved to treat certain types of breast [249], pancreatic [250], gastrointestinal [251], lung [252], kidney cancers [253, 254] and astrocytoma [255]. However, whether everolimus (or other senomorphic drugs) behave as a senomorphic drug in cancer patients has not been evaluated to date.

## The “one‐two punch” approach: combining cancer therapies with senolytics

**Fig. 1:**
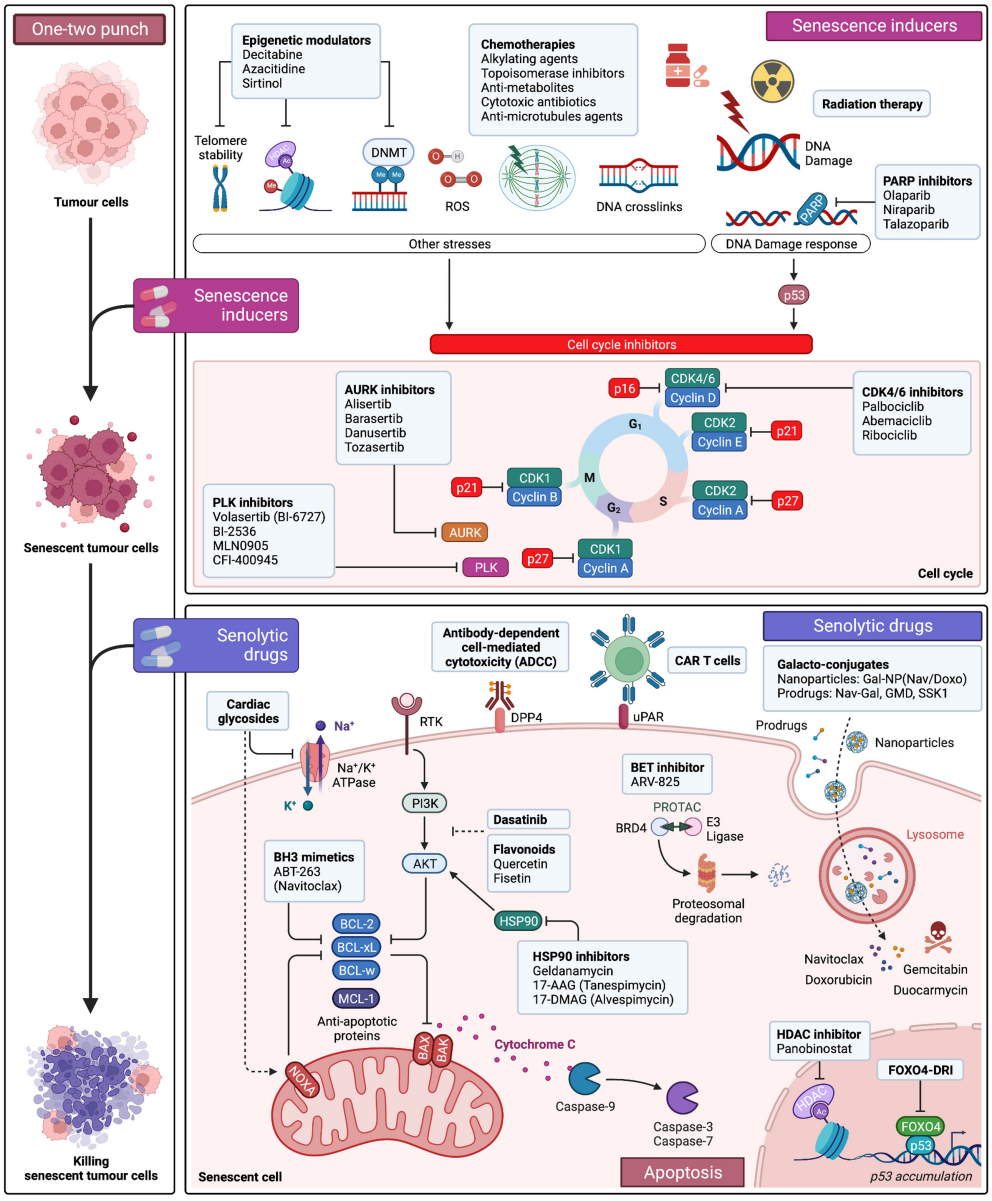
Strategies to target senescence in cancer (“one‐two punch”). Anti‐cancer therapy (first punch) induces the senescence of tumour cells. Conventional chemotherapy, radiotherapy and targeted therapies can be used to induce senescence. Induction of senescence in tumour cells unveils novel targetable vulnerabilities. Senolytic drugs can then be deployed to selectively target and kill senescent tumour cells (second punch). Several senolytic drugs have been described so far and their mechanisms of action are summarised here. Selective killing of senescent cancer cells will improve tumour control and regression. This “one‐two punch” approach to cancer therapy relies on the precise schedule of the administration of the sequential drug treatment to sensitise tumour to senolysis and restore tissue integrity. 17‐AAG, 17‐allylamino‐17‐demethoxygeldanamycin; 17‐DMAG, 17‐dimethylaminoethylamino‐17‐demethoxygeldanamycin; ac, acetylation; ADCC, antibody‐dependent cell‐mediated cytotoxicity; AURK, Aurora kinase; BET, bromodomain and extraterminal domain; CAR‐T cell, chimeric antigen receptor T cell; CDK, cyclin‐dependent kinase; DNMT, DNA methyltransferase; Doxo, doxorubicin; DRI, D‐retro inverso; gal‐NP, galactose‐nanoparticle; GMD, galactose‐modified duocarmycin; HDAC, histone deacetylase; HSP, heat shock protein; Nav, navitoclax; PARP, poly(ADP‐ribose) polymerase; PI3K, phosphatidylinositol tri‐phosphate; PLK, polo‐like kinase; ROS, reactive oxygen species; RTK, receptor tyrosine kinase; uPAR, urokinase plasminogen activator receptor. Created with BioRender.com.

The extension of lifespan and healthspan observed upon genetic ablation of senescent cells in the INK‐ATTAC mice [198] fuelled the interest in developing senolytic drugs, initially aimed to treat an array of age‐related diseases. The potential that eliminating senescent cells have as an anticancer therapy became evident already in these early studies, as the expansion of lifespan observed in the INK‐ATTAC mice is mostly explained by a delay in cancer‐related deaths. While senescent cells influence many aspects of tumour progression, a way to deploy senotherapeutics for cancer treatment is the so‐called “one‐two punch” approach [256]. The rationale of “one‐two punch” therapies is that many cancer therapies induce senescence and using senolytics (as a second punch) would therefore target a newly exposed vulnerability in the cancer cells (Fig. 1).

On 36 breast cancer patients who received adjuvant chemotherapy, te Poele et al. [79] found that 41% of the tumours were SA‐β‐galactosidase and p16 positive, where only 10% of the 20 patients who did not receive chemotherapy stained positive, confirming that cytotoxic chemotherapy induces senescence in patients. Another demonstration comes from Roberson et al. [50] who showed that lung tumours from patient treated with neoadjuvant chemotherapy present large area of SA‐β‐galactosidase positive cells compared to tumours from a patient treated with surgery alone. This also illustrates the heterogeneity of tumour responses to therapy. Given that lingering senescent cells can promote tumour growth, metastasis and therapy resistance, strategies that target the elimination of senescent tumour cells post‐therapy can have multiple benefits.

One of the first demonstrations of the “one‐two punch” concept came from cell culture experiments in which treating A549 cancer cells with Aurora kinase inhibitors (such as alisertib or barasetib) were shown to undergo senescence [131]. Subsequent treatment with ABT‐263 killed the senescent cancer cells, suggesting the validity of such a combination. Given that cancer cells with their different origins and genetic composition might present different vulnerabilities, the Bernards' group concentrated in finding pro‐senescence therapies and senolytics tailored to HCC. Inhibition of CDC7, a DNA replication kinase, with XL413 selectively induces the senescence of p53 mutant cells, while inhibitors of mTOR signalling killed the senescent liver cancer cells [147]. Another example of the one‐two punch treatment is the effective combination of the PARPi olaparib and the senolytic drug navitoclax in a mouse model of ovarian cancer [142]. After a successful demonstration *in vivo*, the authors plan to launch in the mid‐2022 the first phase 1 clinical trial, evaluating the safety and the recommended dose for combined olaparib + navitoclax treatment in ovarian cancer patients, with intermittent delivery of navitoclax to prevent its hematologic toxicity. A future phase 2 study will evaluate the one‐two punch approach in solid tumour, ovarian cancer (NCT05358639).

One‐two punch protocols have been tried with a wide range of other senolytics (Table 3), including cardiac glycosides [211], BRD4 inhibitors [215], and galacto‐coated nanoparticles loaded with doxorubicin or navitoclax [108] or the Gal‐Nav prodrug [213]. There are several clinical trials evaluating the effect of the senolytic Navitoclax in combination with chemotherapy in cancer patients. However, the contribution of senescence and senolysis to the therapeutic effect will not be evaluated on most of those trials (NCT05358639 is an exception, Table 4). In addition, other senolytics, such as D + Q or fisetin, are being evaluated in different trails, including one aiming to improve frailty in adult survivors of childhood cancer (NCT04733534, Table 4). In addition to clinical trials, retrospective analysis is another way to test the potential of drugs repurposed as senolytics. For example, cancer patients treated with the cardiac glycoside digoxin during chemotherapy have a better overall survival [257]. Cardiac glycosides have pleiotropic effects, and the aforementioned study attributed the effect to immunogenic cell death. But given that cardiac glycosides have senolytic properties [209, 211], it would be worthy investigating whether senolysis might explain those results. Finally, a question that remains is what the best timeline for senotherapy is in combination with tumour therapy to achieve maximal benefit.

**Table 4:** Examples of clinical trials testing senolytic drugs in the context of cancer.

Senolytic drug	CT phase	Cancer	Senescence induction	CT number
Navitoclax	Phase 1	Small cell lung cancer	Etoposide, cisplatin	NCT00878449
Navitoclax	Phase 1	Solid tumours	Erlotinib, irinotecan	NCT01009073
Navitoclax	Phase 1	Solid tumours	Gemcitabine	NCT00887757
Navitoclax	Phase 1	Solid tumours	Paclitaxel	NCT00891605
Navitoclax	Phase 1/2	Solid tumours	Trametinib	NCT02079740
Navitoclax	Phase 1	Solid tumours	Docetaxel	NCT00888108
Navitoclax	Phase 1/2	Solid tumours	Dabrafenib, trametinib	NCT01989585
Navitoclax	Phase 1	Advanced or metastatic non‐small cell lung cancer	Osimertinib	NCT02520778
Navitoclax	Phase 1	High‐grade serous carcinoma, triple‐negative breast cancer	Olaparib	NCT05358639
Navitoclax	Phase 1	Myeloid Neoplasms	Decitabine	NCT05455294
Navitoclax	Phase 1	Refractory acute myeloid leukaemia	Decitabine	NCT05222984
Dasatinib and quercetin / Fisetin	Phase 2	Childhood cancer survivors	N/A	NCT04733534

## Effects of senolytics beyond the tumour

Different types of senescent cells are present in the TME during cancer progression and treatment. In addition, cancer treatments are also able to induce senescence in normal tissues. While the use of senolytics as anticancer treatment might be first deployed in the context of “one‐two punch” regimes, senolytics might have benefits that extend beyond killing senescent cancer cells.

OIS, occurring in preneoplastic lesions is a tumour suppressor mechanism that limits tumour progression. Senolytic drugs, such as CGs, have the ability of eliminating preneoplastic senescent cells [209], and therefore might not only target the senescent tumour cells but also limit cancer initiation. Senescent cells (such as fibroblasts) present in the tumour microenvironment are known to promote tumour growth, in part by secreting a pro‐tumorigenic SASP [64]. We and others have shown that strategies that inhibit this pro‐inflammatory SASP decrease tumour growth [12, 13, 258]. Similarly, senolytics such as procyanidin C1 have been proposed to inhibit tumour growth by targeting pro‐tumorigenic fibroblasts in the TME [230]. Moreover, side effects of chemotherapies have been linked to induction of senescence outside the tumour [37]. Using a mouse model for genetic ablation of senescent cells, DeMaria et al. [37] showed how killing senescent cells after chemotherapy reduced bone marrow suppression, cardiac dysfunction, cancer recurrence and improved physical activity and strength. Therefore, an additional advantage of using senolytics for cancer treatment will be limiting the side effects of cancer therapies.

Besides these additional benefits of senolytics we need to consider their side effects. One of the attractions of senolytics is that the original studies suggested an absence of side effects associated with eliminating senescent cells other than delayed wound healing [37, 198, 199]. However, since cancer often affects elderly people, and ageing is associated with slowed wound‐repair and healing abilities [259], delayed wound healing might be problematic. In addition, senolytics such as navitoclax present toxicities associated not with the elimination of senescent cells but with the specific drug target. These drug‐specific toxicities (such as thrombocytopenia caused by navitoclax) also need to be evaluated. In addition, recent studies suggest that, in a way that might depend on the senolytic regime used, elimination of certain senescent cells such as liver sinusoidal endothelial cells (LSECs) [260] can cause additional side effects such as fibrosis. Therefore, the short‐ and long‐term side effects of senolytics remain unknown and need to be considered carefully.

## Future challenges

Senolytics are a relatively novel class of drugs and clinical trials for senolytics are just in their infancy. Senolytics hold immense promise, not just for cancer treatment, but also as therapies for a wide range of age‐related diseases and to treat multimorbidity. However, there are many unknowns and challenges that future research and ongoing trials will help us understand.

There are multiple therapeutics, both novel and clinically approved with senolytic activity and they target different mechanisms. Senolytics include not only small molecules, but also cell and immunotherapies. While this is potentially a very good starting point, it also comes with the added question of which drugs are better suited for which indications. In the case of “one‐two punch” approaches for anticancer therapies, an additional issue is to establish the best regime: when to treat with the senescence‐inducing drug and when with the senolytic? While the rationale argues for sequential treatment (first inducing senescence and then treating with the senolytic), in real life, the heterogeneity of the senescence response in the tumour might need concurrent treatment or a more complicated regime. That of course increases the potential for drug interactions and toxicities that would need to be monitored and managed.

While using senolytics in the context of ‘one‐two punch’ approaches is been postulated as the most clear anticancer therapies, we should not forget about senotherapies that could reduce the pro‐tumorigenic effects associated with the SASP, or ‘reprogram’ the SASP to potentiate anticancer immune surveillance.

Senescence is a cell stress response that is evoked in response to many cancer drugs and in multiple cancer types. While this implies a potentially universal use of senolytics as anticancer therapies, it also opens the question of what are the best cancer types in which start testing the “one‐two punch paradigm”. While pre‐existing data from preclinical models and patients can serve to suggest reasonable starting points, a key element to make senolytic therapies to work is being able to monitor senescence. Identification of senescent cells is one of the issues that has slowed progress in the senescent field for years, even if recent approaches are in development to detect senescence especially in tumours [261]. The issue becomes even more urgent if we want to move senotherapeutics for the clinic. Detection of senescence should be key to enrol and stratify patients and to monitor the efficiency of therapeutic strategies.

To this extent, we ideally need to identify biomarkers that can be routinely surveyed (in plasma or urine) or imaging techniques that could allow a non‐invasive, longitudinal monitoring of senescence in patients. Secreted proteins into biological fluids are routinely used for the surveillance of cancer. Proteins secreted by senescent cells as part of the SASP represent a promising source of biomarkers that could be exploited. For example, a SASP signature has been proposed as an indicator of age and medical risk [262]. Another soluble senescence markers that could be detected in blood include a soluble form of uPAR (suPAR) that is cleaved and secreted by senescent cells and has been used as a biomarker of several senescence‐associated diseases [241]. Interestingly, a study of the lipids present in senescent cells identified the intracellular prostaglandin dh‐15d‐PGJ2 and other prostaglandin D2‐related lipids that accumulate during senescence. Moreover, the dihomo‐prostaglandin is released upon senolysis and thus represents a potential marker of senolysis [263].

The ideal goal would be to monitor the senescence by non‐invasive imaging. While no such an approach is currently used in the clinic, several are in development. For example, [^18^F]FPyGal is a PET tracer aimed to report β‐galactosidase [264, 265]. Activity of β‐galactosidase is often used as a marker of senescence. The [^18^F]FPyGal tracer uptake is increased in senescent cells. [^18^F]FPyGal can reveal the presence of senescence in tumours and was initially evaluated in a patient treated with alisertib, revealing the presence of liver metastasis [264, 265]. Consequently, a clinical trial (NCT04536454) was initiated to further evaluate [^18^F]FPyGal to image senescence in cancer patients.

Overall senotherapies are a very interesting nascent field. While most of the focus has been placed on using senolytics for age‐related diseases, we anticipate that the next years will also see an increase in the use of senolytics in anticancer therapies. The discovery of novel therapeutics and the establishment of protocols to deploy them effectively, combined with strategies to monitor senescence, will all be necessary steps in this process.

## Conflict of interest

JG has acted as a consultant for Unity Biotechnology, Geras Bio, Myricx Pharma and Merck KGaA. Unity Biotechnology and Pfizer have funded research in JG's laboratory. JG owns equity in Geras Bio. JG is a named inventor in MRC and Imperial College patents related to senolytic therapies.

## Author contributions

JG and LB discussed the organisation of the manuscript. LB produced a first draft of the manuscript that was revised and modified by JG and LB.

## Data Availability

There is no data associated with this review.
